# Metabolomics profiling in prediction of chemo-immunotherapy efficiency in advanced non-small cell lung cancer

**DOI:** 10.3389/fonc.2022.1025046

**Published:** 2023-01-17

**Authors:** Lihong Mei, Zhihua Zhang, Xushuo Li, Ying Yang, Ruixue Qi

**Affiliations:** ^1^ Department of Dermatology, Jinshan Hospital, Fudan University, Shanghai, China; ^2^ Department of Echocardiography, Jinshan Hospital, Fudan University, Shanghai, China; ^3^ Center for Tumor Diagnosis and Therapy, Jinshan Hospital, Fudan University, Shanghai, China

**Keywords:** chemo-immunotherapy, non-small cell lung cancer, metabolomics, biomarker, GC-MS

## Abstract

**Background:**

To explore potential metabolomics biomarker in predicting the efficiency of the chemo-immunotherapy in patients with advanced non-small cell lung cancer (NSCLC).

**Methods:**

A total of 83 eligible patients were assigned to receive chemo-immunotherapy. Serum samples were prospectively collected before the treatment to perform metabolomics profiling analyses under the application of gas chromatography mass spectrometry (GC-MS). The key metabolites were identified using projection to latent structures discriminant analysis (PLS-DA). The key metabolites were used for predicting the chemo-immunotherapy efficiency in advanced NSCLC patients.

**Results:**

Seven metabolites including pyruvate, threonine, alanine, urea, oxalate, elaidic acid and glutamate were identified as the key metabolites to the chemo-immunotherapy response. The receiver operating characteristic curves (AUC) were 0.79 (95% CI: 0.69-0.90), 0.60 (95% CI: 0.48-0.73), 0.69 (95% CI: 0.57-0.80), 0.63 (95% CI: 0.51-0.75), 0.60 (95% CI: 0.48-0.72), 0.56 (95% CI: 0.43-0.67), and 0.67 (95% CI: 0.55-0.80) for the key metabolites, respectively. A binary logistic regression was used to construct a combined biomarker model to improve the discriminating efficiency. The AUC was 0.86 (95% CI: 0.77-0.94) for the combined biomarker model. Pathway analyses showed that urea cycle, glucose-alanine cycle, glycine and serine metabolism, alanine metabolism, and glutamate metabolism were the key metabolic pathway to the chemo-immunotherapy response in patients with advanced NSCLC.

**Conclusion:**

Metabolomics analyses of key metabolites and pathways revealed that GC-MS could be used to predict the efficiency of chemo-immunotherapy. Pyruvate, threonine, alanine, urea, oxalate, elaidic acid and glutamate played a central role in the metabolic of PD patients with advanced NSCLC.

## Introduction

The incidence rate of lung cancer is the highest worldwide, the five-year survival rate of which is lower than 20% (1). Non-small cell lung cancer (NSCLC) including lung adenocarcinoma, squamous cell carcinoma and large cell carcinoma account for about 40% of lung cancers (2). Options for the treatment of NSCLC include surgery, radiotherapy, chemotherapy, targeted therapy and immunotherapy, or a combination of these treatments (3).

The mechanism of standard chemotherapy is to act directly on cancer cells to induce tumour lethality. The main effect of immunotherapy including anti-programmed death-1 (PD-1) or anti-programmed death-ligand 1 (PD-L1) is to enhance CD8+ cytotoxic T-cell immune response with the aim of reinvigorating the immune system against cancer cells (4). Recently, the combination of immunotherapy with standard chemotherapy to enhance their mechanism of action has shown benefits in phase III randomized control trials (5). However, to select the optimal patient for chemo-immunotherapy remains a challenge. Improper selection of patients would lead to under- or over-treatment.

The tumor micro-environment involves complex interaction between intracellular and extracellular metabolites, which would affect the anti-tumour efficiency of chemo-immunotherapy (4, 6). Metabolism has been proposed as a mechanism potentially affecting the response and the toxicity to immunotherapy and chemotherapy (7, 8). Metabolomics is an omics technology reflecting a disease state, which makes metabolomics a valuable field for monitoring disease status and exploring biomarkers to predict the efficiency of therapy (9). Ghini et al. showed that high serum alanine and pyruvate in non-responder of immunotherapy in NSCLC using nuclear magnetic resonance spectroscopy metabolomics (8). Miller et al. found that high pyruvate and uric acid in non-responder of chemotherapy in NSCLC tumor tissues using liquid chromatography-mass spectrometry metabolomics (10).

However, to our knowledge, no study reported potential metabolism biomarkers that related to the efficiency of chemo-immunotherapy in advanced NSCLC. We hypothesize that specific serum metabolic phenotype could be used to predict the non-responders to chemo-immunotherapy. To explore this hypothesis, we used gas chromatography mass spectrometry (GC-MS) to perform a metabolomics profiling analysis to identify potential metabolic biomarkers to predict the efficiency of chemo-immunotherapy in patients with advanced NSCLC.

## Methods

### Study design and patient population

This prospective study was reviewed by the Institutional Review Board of Jinshan Hospital, Fudan University (No.JIEC2021S47). All patients signed the informed consent. All the methods were carried out in accordance with relevant guidelines and regulations.

From December 2018 to October 2021, 97 consecutive patients were enrolled in this study. Inclusion criteria (1): patients with pathological confirmed NSCLC with advanced stage IV (TxNxM1); (2) patients received toripalimab (240 mg) plus cisplatin-paclitaxel (75 mg/m^2^ and 260 mg/m^2^) for 4-6 cycles followed by toripalimab (240 mg) monotherapy until unacceptable toxicity or disease progression; (3) patients with negative EGFR, ALK, ROS1, MET mutation. Exclusion criteria: (1) patients with symptoms associated with infection, such as increased leukocytes and neutrophils, or inflammation indicated by lung CT (n = 5); (2) patients not completing 4 cycles of chemo-immunotherapy (n = 4); (3) patients lost of follow-up (n = 5). Finally, 83 patients were enrolled in the further analysis. They were deviled into a disease control (DC) group (including complete response [CR], partial response [PR] and stable disease [SD]) and a progressive disease (PD) group. The tumor responses to the chemo-immunotherapy were evaluated by chest CT according to Response Evaluation Criteria in Solid Tumors, version 1.1 (RECIST v1.1) (11).

### Clinical features and serum sample collection

Clinical features including age, gender, tumor position, metastases position, pathological sub-type, and PD-L1 expression were collected. Fasting peripheral blood (2-4 mL) was collected with a serum separator tube within one week before the first cycle of chemo-immunotherapy. The patients were not receiving other drug therapies when sampling before chemo-immunotherapy. The blood was centrifuged at 1,200 rpm for 10 min at 4°C within 30 min after collection, and stored at -80°C.

### Metabolite extraction and profiling analysis

Serum samples (50 µL) were thawed and vortexed thoroughly on ice. Serum sample was mixed with 250 µL methanol aqueous (4:1) solution containing tridecanoic acid 5 µg/mL. The mixture was vortexed for 30 s and incubated at 37°C, vortexed at 1,200 rpm for 30 min, and centrifuged at 14,000 rpm at 4°C for 15 min. 225 µL supernatant was transferred to a fresh tube, vortexed with water for 30 s and centrifuged at 14,000 rpm at 4°C for 5 min. 250 µL supernatant was transferred to a fresh tube and dried. The dried samples were reconstituted in 50 µL methoxyamine hydrochloride, ultrasonic vibration 1 min, vortexed at 1,200 rpm at 30°C for 90 min. 40 µL MSTFA was added in the mixture and vortexed at 1,200 rpm at 37°C for 30 min. The mixture was centrifuged at 12,000 rpm at 4°C for 5 min. Finally, 60 µL of supernatant was used for GC-MS analysis. The analyses were performed on GC-MS (7890B-5977A, Agilent Technologies, Waldrom, Germany) (12). The column and injection port temperatures were 50 C and 280 C, respectively. The temperature was held at 50 C for 2 min, increased at 5 C/min to 180 C, held for 5 min, and then increased at 10 C/min to 290 C, and held for 3 min. The carrier gas was high-purity helium (99.999%), the fow rate was 1 mL/min, the pre-column pressure was 69.8 kPa, the split ratio was 10:1, and the injection volume was 1.0 µL. Mass spectrometry conditions: electron ionization ion source 230 C, solvent delay 3 min, electron energy 70 eV, quadrupole temperature 150 C, scanning range 40-500 m/z, multiresolution signal decomposition transmission line temperature 230 C, and electron multiplier voltage 2,300 V. The Agilent Fiehn database was referred to for identification of the metabolites. By using the formula: standard sample peak area/standard sample concentration = sample peak area/sample concentration, the metabolites content in the sample was calculated (13).

### Data processing

First, metabolites with at least 80% samples in both group were retained. Second, the remaining missing values were replace by median. Third, the data was normalized by median and scaled by auto scaling (mean-centered and divided by the standard deviation of each variable).

Unsupervised analysis (principal component analysis, PCA) was applied to explore the clustering patterns and outliers between the groups. Supervised analysis (projection to latent structures discriminant analysis, PLS-DA) was used for pattern recognition analysis of the key metabolites in DC and PD group. The metabolites with variable influence on projection (VIP) score > 1.5 and P < 0.05 were defined as the key metabolites. A correlation heatmap was computed to analyze the correlation between the metabolomics features and the clinical features.

### The metabolites’ discrimination between DC and PD

The receiver operator characteristic (ROC) and area under the ROC curve (AUC) were employed to evaluate the diagnostic performance of the key metabolites in predicting non-responders of chemo-immunotherapy. A binary logistic regression was used to construct a combined biomarker model to improve the discriminating efficiency.

### Enrichment and pathway analysis

Enrichment and pathway analysis were performed to encompasses the key metabolites from different biological pathways that generate a biological perturbation. The top-ranked pathways related to the key metabolites were represented based on the KEGG database (https://www.kegg.jp/).

### Survival analysis in DC and PD

The progress free survival (PFS) and overall survival (OS) curves of PD and DC groups were plotted. The overall 3-year survival rate in advanced NSCLS patients was shown.

### Statistical analysis

The statistical analyses were performed using MetaboAnalyst (v5.0, https://www.metaboanalyst.ca/) and R (v4.2.0, https://www.r-project.org/). Shapiro-Wilk test and Bartlett test were used to assess the normality and variance homogeneity. The key metabolites and clinical features were compared by T-test (met normality and variance homogeneity), Mann-Whitney U test (if not met normality or variance homogeneity) followed by false discovery rate (FDR) or by Chi square test (if they were categorical variables). Survival analysis was assessed with Kaplan-Meier curves *via* log-rank tests. The ROC and AUC were employed to evaluate the predictive performance of the key metabolites. All the statistical tests were two-sided and considered statistically significant at P < 0.05.

## Results

### Patient baseline characteristics

**Figure 1:**
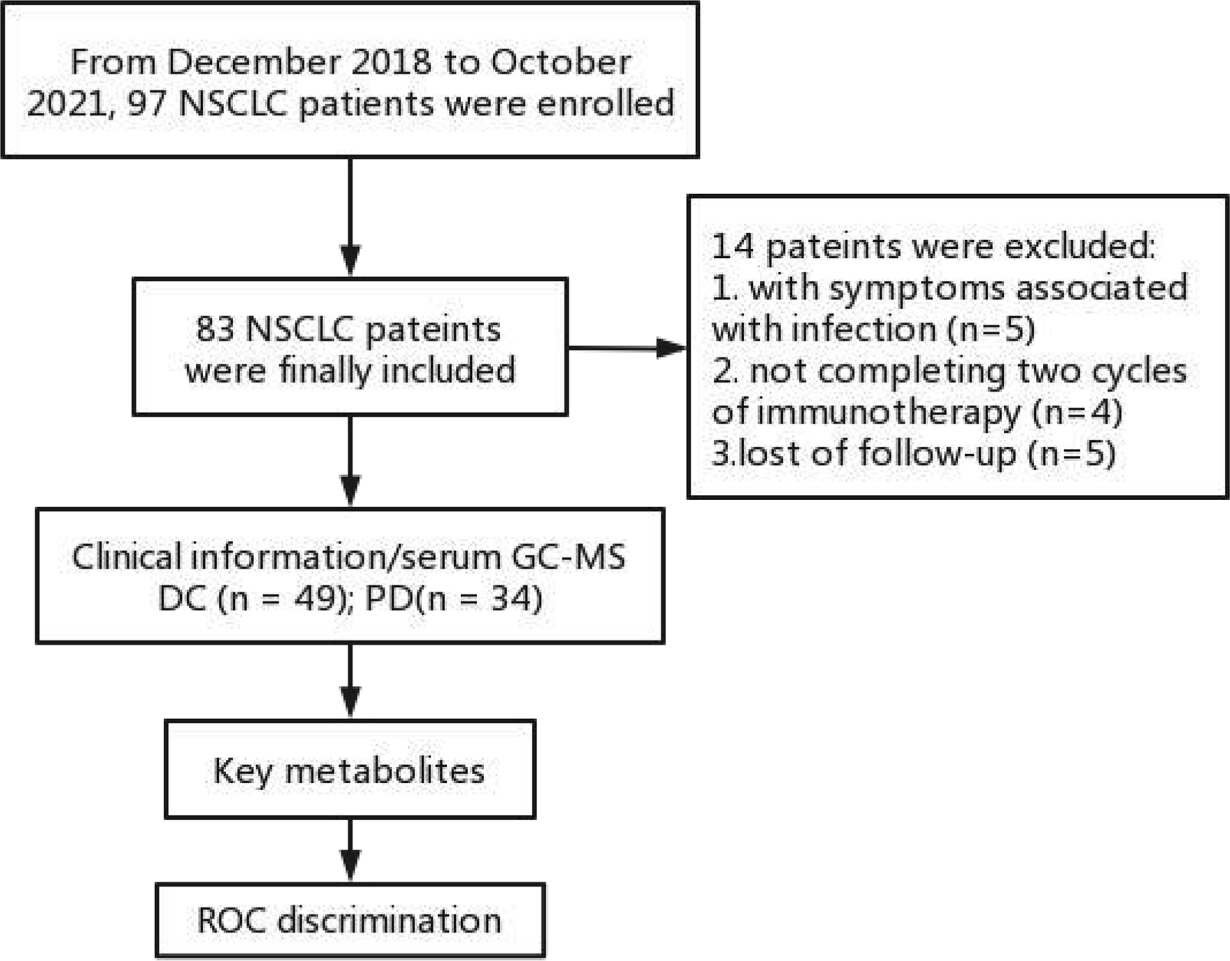
Work flow of this study. DC, disease control; ROC, receiver operating characteristic curve; PD, progressive disease.

In total, serum samples of 83 patients were collected from the patients with histologically confirmed NSCLC. Of these patients (median age 64; ranged 44-83), 49 patients (median age 62; ranged 54-83) were enrolled in the DC group and 34 patients (median age 65; ranged 50-81) were enrolled in the PD group. The work flow of this study is shown in **Figure 1**. All the patients received chemo-immunotherapy as the first-line therapy. Most patients achieved disease control, including those with a confirmed CR (n = 0), PR (n = 9) and SD (n = 40), respectively. The clinical characteristics are shown in **Table 1**. No significant differences of the gender, tumor position, metastases position, pathological sub-type, and PD-L1 expression were shown between DC group and PD group. Older age, and shorter PFS and OS were shown in PD group compared with DC group (**Table 1**).

**Table 1:** Comparison of clinical characteristics between DC and PD patients.

	DC (N=49)	PD (N=34)	P
Age (y)	61 (7.9)	66 (8.3)	0.020
Tumor size (mm)	49.8 (20.5)	47.4 (25.8)	0.649
Gender			
Female	14 (28.6%)	10 (29.4%)	1
Male	35 (71.4%)	24 (70.6%)	
Tumor position			
Central lung cancer	10 (20.4%)	5 (14.7%)	0.708
Peripheral lung cancer	39 (79.6%)	29 (85.3%)	
Metastases position			
Lung			
Negative	34 (69.4%)	21 (61.8%)	0.627
Positive	15 (30.6%)	13 (38.2%)	
Brain			
Negative	32 (65.3%)	24 (70.6%)	0.790
Positive	17 (34.7%)	10 (29.4%)	
Bone			
Negative	35 (71.4%)	24 (70.6%)	1
Positive	14 (28.6%)	10 (29.4%)	
Liver			
Negative	40 (81.6%)	29 (85.3%)	0.889
Positive	9 (18.4%)	5 (14.7%)	
Other			
Negative	44 (89.8%)	29 (85.3%)	0.782
Positive	5 (10.2%)	5 (14.7%)	
Pathological sub-type			
Adenocarcinoma	27 (55.1%)	19 (55.9%)	0.177
Large cell carcinoma	4 (8.2%)	7 (20.6%)	
Squamous cell carcinoma	18 (36.7%)	8 (23.5%)	
Status			
Survival	38 (77.6%)	17 (50.0%)	0.018
Dead	11 (22.4%)	17 (50.0%)	
PD-L1 expression			1
< 50%	18 (36.7%)	13 (38.2%)	
> 50%	31 (63.3%)	21 (61.8%)	
Group			
CR	0 (0%)	0 (0%)	<0.001
PR	9 (18.4%)	0 (0%)	
SD	40 (81.6%)	0 (0%)	
PD	0 (0%)	34 (100%)	

CR, complete response; DC, disease control; PD, progressive disease, PR, partial response. Data presented as mean (SD) or N (ratio).

### Metabolomics profiling of pre-chemo-immunotherapy serum samples

**Figure 2:**
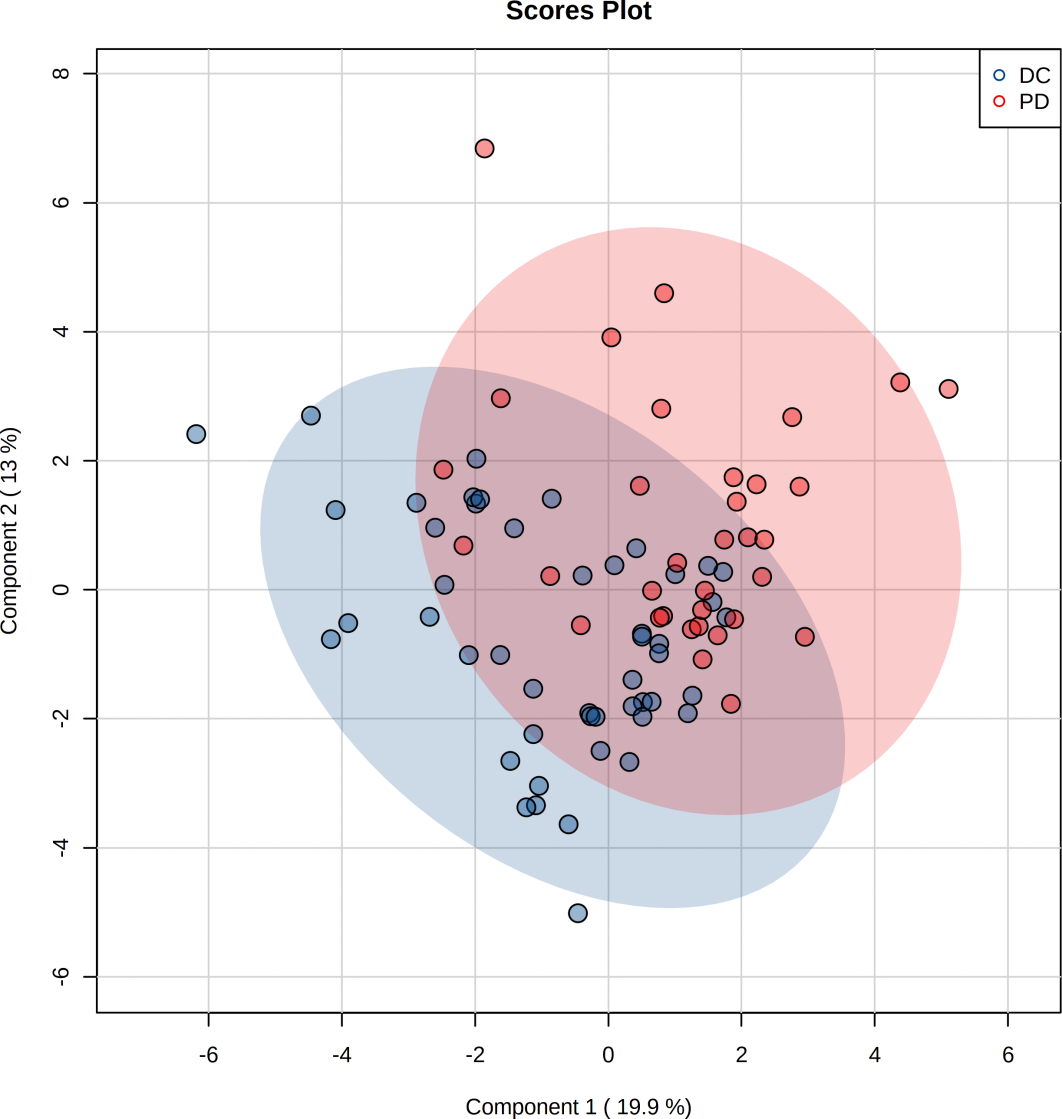
Projection to latent structures discriminant analysis (PLS-DA) of the metabolites in DC and PD groups. The metabolites between DC and PD groups are well separated by PLS-DA.

**Figure 3:**
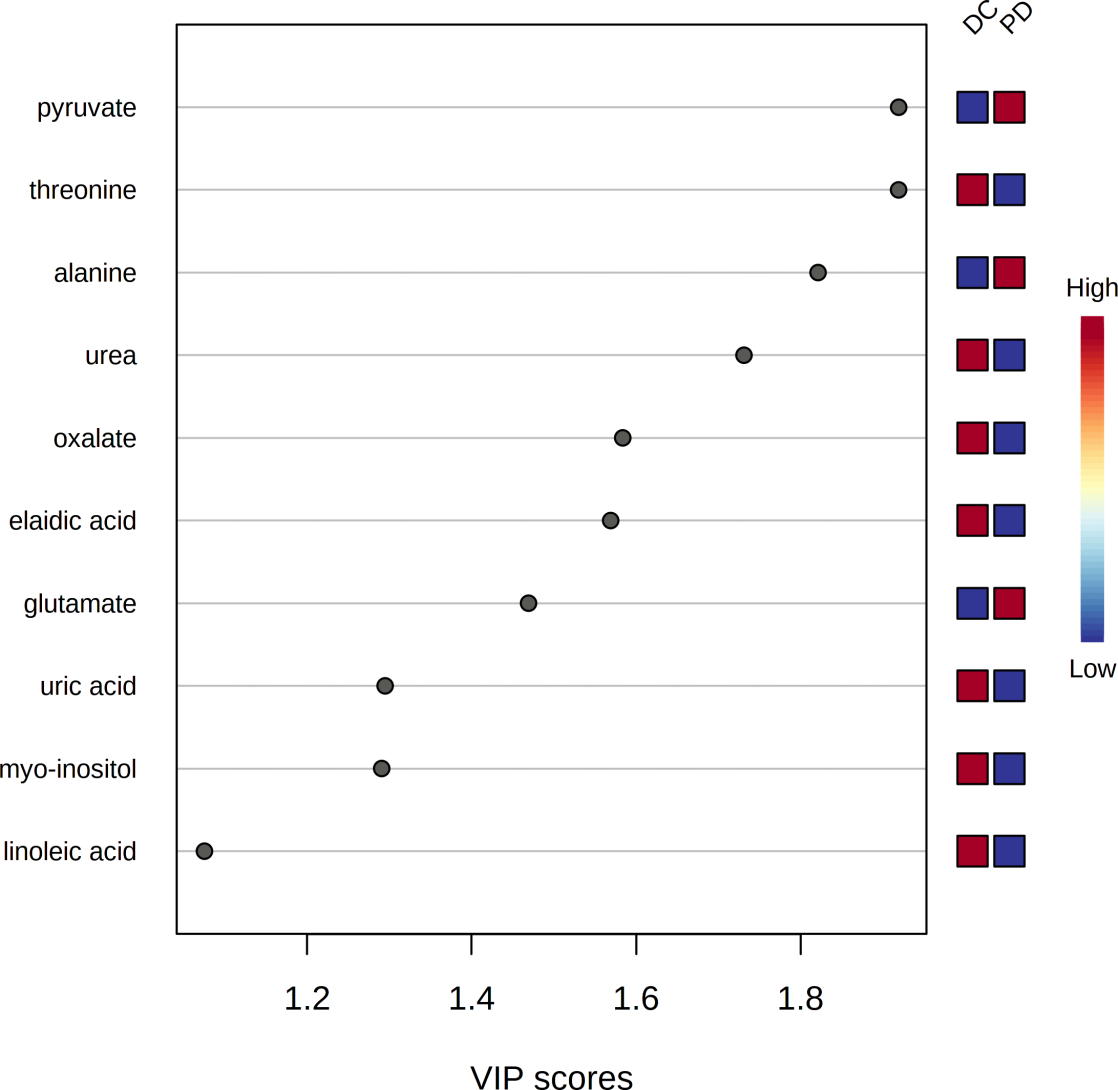
The variable influence on projection (VIP) score of the metabolites by PLS-DA. Seven metabolites including pyruvate, threonine, alanine, urea, oxalate, elaidic acid and glutamate were identified as the key metabolites to the chemo-immunotherapy response.

**Figure 4:**
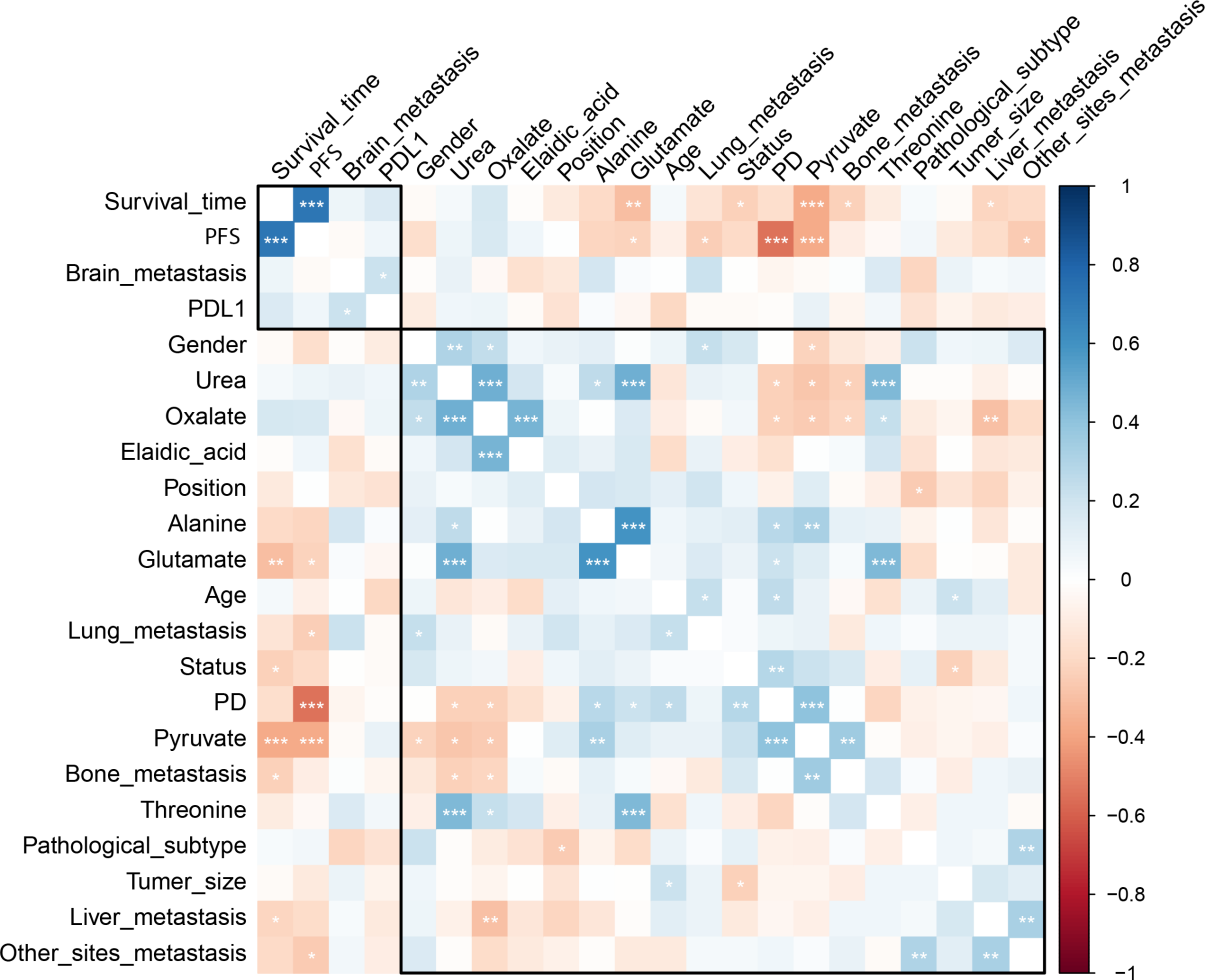
The correlation heatmap shows the correlation of the key metabolites and the clinical features. Significantly negative correlation was found between the level of pyruvate and OS as well as PFS, and between the level of alanine and PFS. *, < 0.05; **< 0.01; ***, < 0.001.

The pre-chemo-immunotherapy serum samples were analysis by using GC-MS. After removal of missing values, 51 metabolites were obtained. PCA plot showed the metabolites between DC group and PD group with QC samples (**Supplementary Figure 1**). The metabolites between DC group and PD group could be separated by PLS-DA scores plots (R^2^ = 0.63, Q^2^ = 0.14) (**Figure 2**). Seven key metabolites were identified between the DC group and the PD group. These metabolites were considered as the subsequent identification of potential biomarkers for predictive of chemo-immunotherapy response in advanced NSCLC (**Figure 3**). In these metabolites, 3 (pyruvate, alanine, and elaidic acid) with higher levels, and 4 (threonine, urea, oxalate, and glutamate) with lower levels were found in the PD group compared to the DC group (**Supplementary Table 1**). A correlation heatmap shows the correlation of the key metabolites and the clinical features (**Figure 4**).

### The metabolites’ discrimination between DC and PD

Seven metabolites including pyruvate, threonine, alanine, urea, oxalate, elaidic acid and glutamate were identified as the key metabolites to the chemo-immunotherapy response. The receiver operating characteristic curves (AUC) were 0.79 (95% CI: 0.69-0.90), 0.60 (95% CI: 0.48-0.73), 0.69 (95% CI: 0.57-0.80), 0.63 (95% CI: 0.51-0.75), 0.60 (95% CI: 0.48-0.72), 0.56 (95% CI: 0.43-0.67), and 0.67 (95% CI: 0.55-0.80) for the key metabolites, respectively. The AUC was 0.86 (95% CI: 0.77-0.94) for the combined biomarker model.

### Enrichment and pathway analysis

Results showed that urea cycle, glucose-alanine cycle, glycine and serine metabolism, alanine metabolism, and glutamate metabolism were evolved in chemo-immunotherapy response in advanced NSCLC patients (**Supplementary Figure 2**).

### Survival analysis in DC and PD

**Figure 5:**
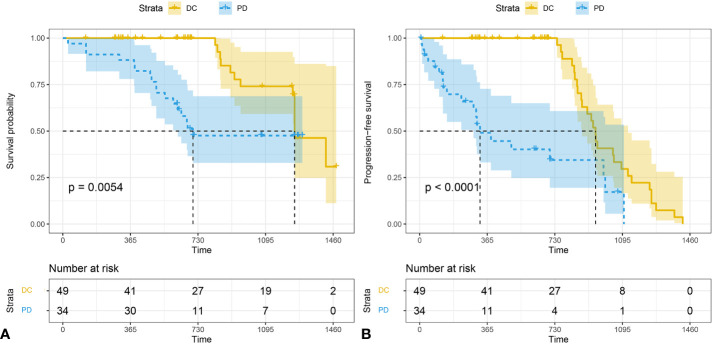
The Kaplan-Meier analysis of prognostic prediction in DC group and PD group. Shown are the analysis of the progression-free survival (median time, 1252 d for DC and 326 d for PD) **(A)**, and the overall survival (median time,1252 d for DC and 674 d for PD) **(B)**. The 3-year survival rates were 38.7% (19/49) for DC group and 20.5% (7/34) for PD group. P values were calculated by log-rank test. DC, disease control; PD, progressive disease.

The median follow-up time for all subject was 696 d (range 28-1477). The median follow-up time was 850 (range 124-1477) for DC group and 655 (range 28-1294) for PD group. The 3-year survival rates were 38.7% (19/49) for DC group and 20.5% (7/34) for PD group. The PFS of patients who were classified as DC cases was significantly longer than that of PD cases (P < 0.001). The OS of DC patients was significantly longer than that of PD patients (P = 0.005) (**Figure 5**).

## Discussion

In this study, GC-MS metabolomics was developed to predict the efficiency of chemo-immunotherapy in patients with advanced NSCLC before treatment delivery. By using metabolomics analysis, pyruvate, threonine, alanine, urea, oxalate, elaidic acid and glutamate were identified as the key metabolites predicting the non-responders of chemo-immunotherapy in NSCLC patients.

Metabolites are low molecular weight products of the cellular processes, which are fundamental to understand the functional status of cells (14). Metabolomics is functional readouts of a cellular state, which can reflect alterations of biological states (15). Thus, metabolomics is more likely to provide candidates of potential biomarkers of diseases (16). Cancer cells metabolism is profoundly altered and, therefore, produces molecules that are specific and typical of non-physiological conditions (14). In consonance with peptide expression as an indicator of disease, the abundance of cancer-driven metabolic abnormalities may be representative of a disease state or be indicative of the cancer’s pathogenesis, allowing for improved diagnostics and disease monitoring (17). The property of metabolites interacting with and targeting of therapeutics makes metabolomics a valuable field for advancements in assessment the efficiency of chemo-immunotherapy.

In this study, a total of 7 metabolites were selected as the predictor of chemo-immunotherapy efficiency in advanced NSCLC. These metabolites are mainly involved in the metabolism of alanine metabolism, glucose-alanine cycle, urea cycle, glycine and serine metabolism, and glutamate metabolism which are closely associated with cancer progression. Previous study reported that higher levels of serum pyruvate, alanine, lactate and glycine were detected in non-responder subjects of immunotherapy in NSCLC (8). Higher levels of pyruvate, alanine, glycine, and lactate indicated an increase of glycolysis. Pyruvate has a central role in glycolysis, citric acid cycle, urea cycle, and amino acid metabolism. Sellers et al. reported that pyruvate was critical for promoting NSCLC cancer proliferation, which was correlated with PD (18). Pyruvate was also reported related to the tumor angiogenesis and the immune response (19). Pyruvate was also reported as a strong predictor of chemotherapy response. Increased serum pyruvate levels in PD patients may indicate an increase in metabolic rate in aggressive NSCLC patients (10). In this study, significantly negative correlation was found between the level of pyruvate and OS as well as PFS, which is in according to the previous studies (8, 10). Furthermore, significantly negative correlation was found between the level of alanine and PFS.

Urea is largely derived from the urea cycle reactions through hepatic detoxification of free ammonia and cleared by urination. Recently, study revealed that an intriguing link between serum urea and cancer risks (20). Previous study reported that serine/threonine kinase 2 promotes proliferation can be used to predict metastasis and poor prognosis in NSCLC (21). The efficiency of epidermal growth factor receptor tyrosine kinase inhibitors for EGFR gene is also well established in NSCLC (22). Park et al. reported that xylitol as anti-caries agent that has anti-inflammatory effects, which has potential in therapy against lung cancer by inhibiting cell proliferation and inducing autophagy of A549 cells (23). Balan et al. Reported that thymol could act as a safe and potent therapeutic agent to treat NSCLC by inducing mitochondrial pathway-mediated apoptosis *via* ROS generation, macromolecular damage and SOD diminution in A549 cells (24). Puchades-Carrasco et al. reported that lower levels of serum glutamine, threonine and histidine in NSCLC patients compared with healthy individuals (25). Zhang et al. reported that the decrease of serum threonine and histidine were attributed to the up-regulation of the glycine/serine/threonine and pyrimidine metabolic pathways (26). Zhao et al. found that the occurrence and development of lung cancer are closely related to disturbance of glutamine and glutamate metabolism (27). An elevation of elaidic acid in PD patients indicated that lipid metabolism was also involved in the response of chemo-immunotherapy.

This study had several limitations. First, the metabolite is highly dynamic and sensitive to a wide range of factors, the result of this study needs to be validated to warrant the consistency and reproducibility. Second, some of the patients were not able for surgical management before the chemo-immunotherapy, which may have potential influence on the results due to selection bias. Furthermore, larger sample, multi-center and prospective studies should be carried out for validating the metabolomics nomogram to provide reliable evidence for further clinical application.

In conclusion, The discriminant metabolomics profiling developed in this study offered a feasible and convenient strategy to personalize treatment. The high accuracy of metabolomics profiling provides the possibility of predicting the effect of chemo-immunotherapy in NSCLC patients.

## Data availability statement

The raw data supporting the conclusions of this article will be made available by the authors, without undue reservation.

## Ethics statement

The studies involving human participants were reviewed and approved by Institutional Review Board of Jinshan Hospital, Fudan University. The patients/participants provided their written informed consent to participate in this study.

## Author contributions

LM and RQ designed the research study. LM, ZZ and RQ performed the research. XL and YY provided help and advice on acquisition of data. LM analyzed the data. LM and RQ wrote the manuscript. All authors contributed to the article and approved the submitted version.
