# Supplementary material for: Metabolomics profiling in prediction of chemo-immunotherapy efficiency in advanced non-small cell lung cancer
Source: Front Oncol. 2023 Jan 17;12:1025046. doi: 10.3389/fonc.2022.1025046 (PMC9887290; doi:10.3389/fonc.2022.1025046)
Supplement: Supplementary file 1 [file DataSheet_1.docx]

Supplementary Table 1. Fold change and P values of the key metabolites (PD/DC)

|  | FC | log2(FC) | P |
| --- | --- | --- | --- |
| Pyruvate | 2.351 | 1.233 | 0.003 |
| Threonine | 0.614 | -0.703 | 0.003 |
| Alanine | 1.740 | 0.799 | 0.005 |
| Urea | 0.653 | -0.615 | 0.008 |
| Oxalate | 0.721 | -0.471 | 0.015 |
| Elaidic acid | 1.402 | 0.4875 | 0.016 |
| Glutamate | 0.825 | -0.276 | 0.024 |


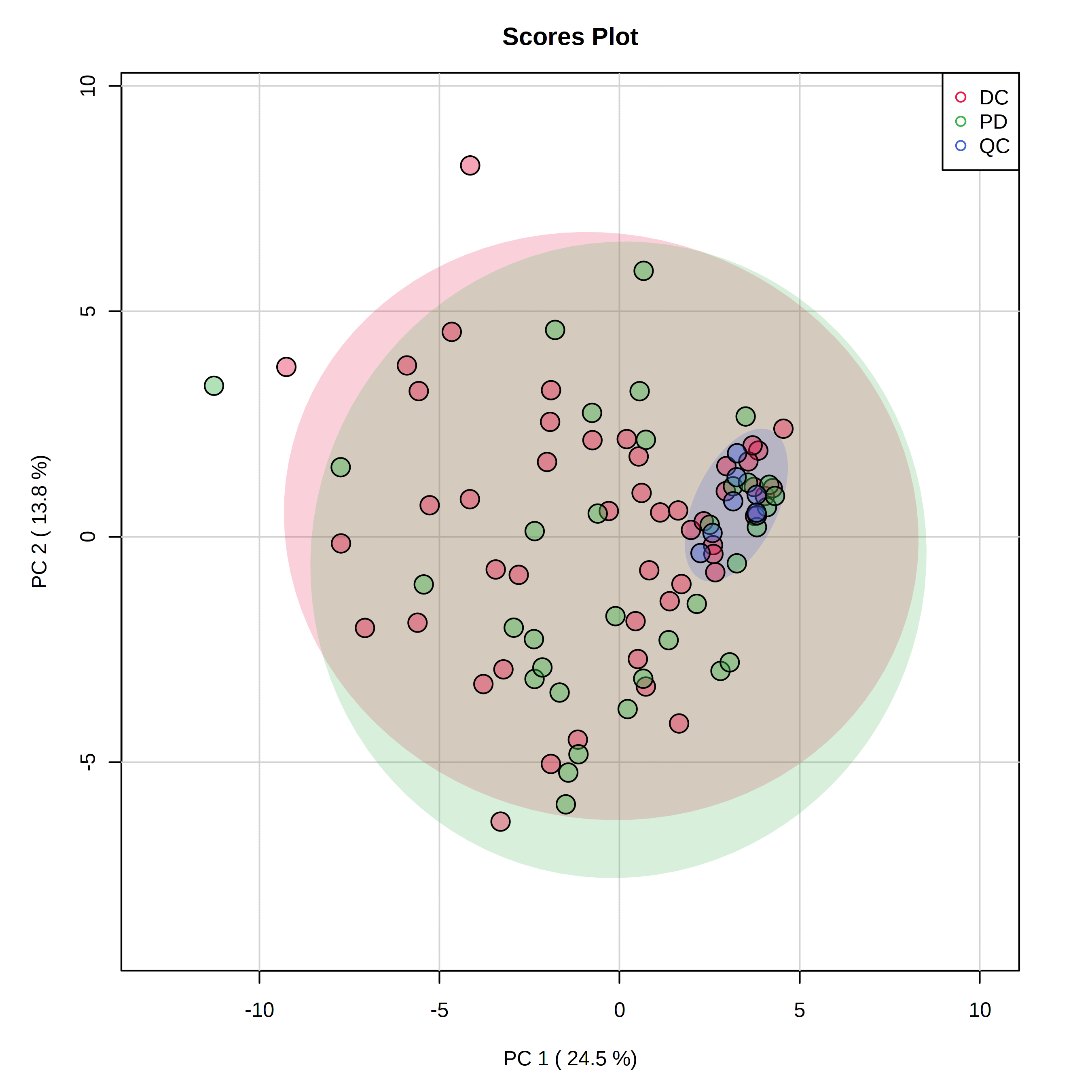


Supplementary Figure 1. The PCA plot of the 51 metabolites of PD and DC group with QC samples.


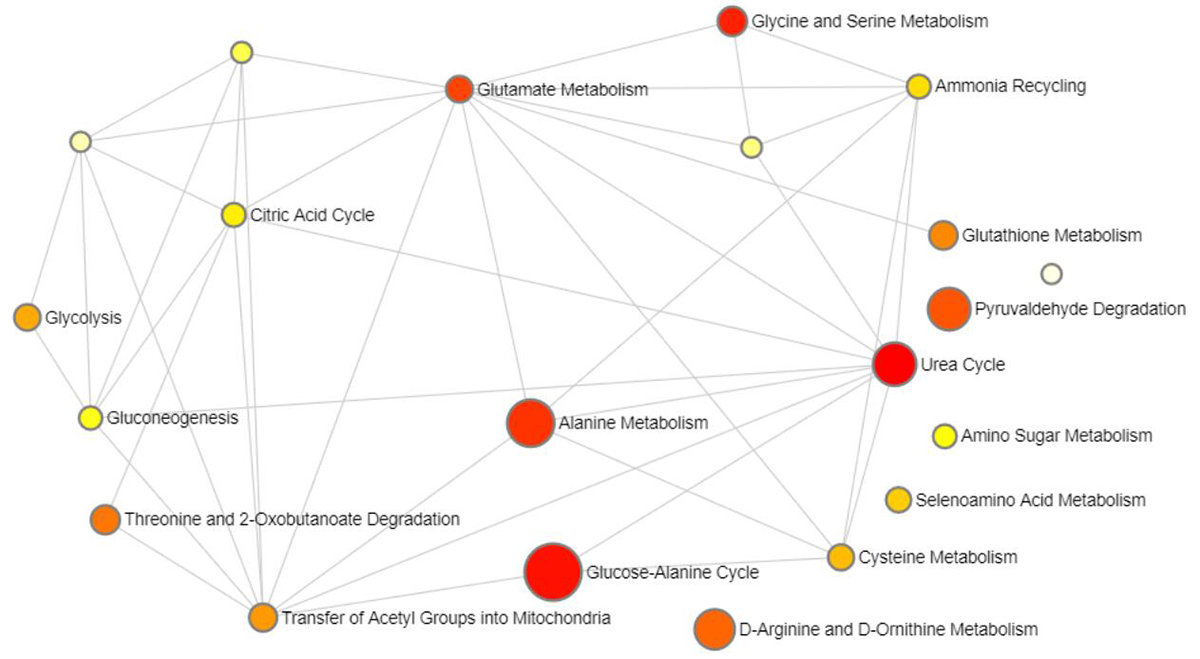


Supplementary Figure 2. The metabolic pathways of the key metabolites involved in predicting the chemo-immunotherapy efficiency in advanced non-small cell lung cancer.
